# New Findings in Cleavage Sites Variability across Groups, Subtypes and Recombinants of Human Immunodeficiency Virus Type 1

**DOI:** 10.1371/journal.pone.0088099

**Published:** 2014-02-07

**Authors:** Esther Torrecilla, Teresa Llácer Delicado, África Holguín

**Affiliations:** HIV-1 Molecular Epidemiology Laboratory, Dept. of Microbiology, Hospital Ramón y Cajal- IRYCIS and CIBERESP, Madrid, Spain; Institut Pasteur, France

## Abstract

**Background:**

Polymorphisms at cleavage sites (CS) can influence Gag and Pol proteins processing by the viral protease (PR), restore viral fitness and influence the virological outcome of specific antiretroviral drugs. However, data of HIV-1 variant-associated CS variability is scarce.

**Methods:**

In this descriptive research, we examine the effect of HIV-1 variants on CS conservation using all 9,028 *gag* and 3,906 *pol* HIV-1 sequences deposited in GenBank, focusing on the 110 residues (10 per site) involved at 11 CS: P17/P24, P24/P2, P2/P7, P7/P1, P1/P6*^gag^*, NC/TFP, TFP/P6*^pol^,* P6*^pol^*/PR, PR/RT^p51^, RT^p51^/RT^p66^ and RT^p66^/IN. CS consensus amino acid sequences across HIV-1 groups (M, O, N, P), group M 9 subtypes and 51 circulating recombinant forms (CRF) were inferred from our alignments and compared to the HIV-1 consensus-of-consensuses sequence provided by GenBank.

**Results:**

In all HIV-1 variants, the most conserved CS were PR/RT^p51^, RT^p51^/RT^p66^, P24/P2 and RT^p66^/IN and the least P2/P7 and P6*^pol^*/PR. Conservation was significantly lower in subtypes *vs.* recombinants in P2/P7 and TFP/P6*^pol^* and higher in P17/P24. We found a significantly higher conservation rate among Group M *vs*. non-M Groups HIV-1. The late processing sites at Gag (P7/P1) and GagPol precursors (PR/RT^p51^) presented a significantly higher conservation *vs.* the first CS (P2/P7) in the 4 HIV-1 groups. Here we show 52 highly conserved residues across HIV-1 variants in 11 CS and the amino acid consensus sequence in each HIV-1 group and HIV-1 group M variant for each 11 CS.

**Conclusions:**

This is the first study to describe the CS conservation level across all HIV-1 variants and 11 sites in one of the largest available sequence HIV-1 dataset. These results could help other researchers for the future design of both novel antiretroviral agents acting as maturation inhibitors as well as for vaccine targeting CS.

## Introduction

The human immunodeficiency virus type 1 (HIV-1) Gag proteins are essential for the virus, as they have a structural and functional role in the viral cycle. They coordinate viral trafficking, membrane binding, assembly, cofactor packaging, budding, and viral modulation. Gag proteins are generated through viral maturation, essential in the viral life cycle by enabling the generation of mature infectious viral particles through the proteolytic process in specific cleavage sites (CS) of Gag precursor (Pr55*^gag^*) and GagPol precursors (Pr160^GagPol^) proteins by the viral protease (PR) [1], [2]. Gag precursor is cleaved within the virion in three main structural Gag proteins: matrix (P17 or MA), capsid (P24 or CA) and nucleocapsid (P7 or NC), flanked by two spacer segments (P1 and P2) with regulatory functions [3]. Gag P6, a sixth protein of Gag precursor, plays an essential role in the release of the virus from infected cell membranes [3]. During translation of the Gag precursor an occasional ribosomal frameshift leads to the production of a GagPol precursor protein, the abundance of which is approximately 5% that of Gag precursor [4]. GagPol precursor contains the main structural proteins matrix P17, P24, P7, a transframe protein (TFP), P6*^pol^* and the three viral replication enzymes, PR, reverse transcriptase (RT) and integrase (IN) [3]. PR is activated concomitant with viral budding. As PR is only active as a dimer, it is thought that autoprocessing is initiated by dimerization of two PR domains that are embedded in the GagPol precursor [5]. Maturation triggers a second assembly event that generates a condensed conical capsid core, which organizes the viral RNA genome and viral proteins to facilitate viral replication in the next round of infection [6].

Processing of both HIV-1 Gag and GagPol polyproteins by the viral PR is highly specific, temporally regulated, and essential for the production of infectious HIV-1 particles. The differential rate of processing at each of the 11 proteolytic reactions by cleavage exists [6] and is determined by the context surrounding processing sites of the CS [7]. However, the precise mechanisms governing the rates of the cleavage events are still not fully understood [7].

The physical consequence of Gag cleavage is a morphological rearrangement of the non-infectious immature particle to a mature infectious particle. For this reason, amino acid substitutions on Gag proteins, included in CS, could influence processing [2], [8], morphogenesis, budding [9], the virus replicative capacity or *viral fitness* [3], [10] and the virological outcome of specific regimens, particularly to protease inhibitors (PI) [5], [11]–[20]. In fact, several Gag substrate mutations, included in CS, can confer PI resistance in the absence and/or presence of PR mutations [17]–[20]. The fundamental role of proteolytic maturation in the generation of infectious particles makes inhibition of this process an attractive target for therapeutic intervention. Thus, a new class of potential antiretroviral drugs targeting individual Gag CS has entered development [21].

Whether or not the processing regulation is different across HIV-1 variants remains unclear. It is well known that HIV-1 shows a high genetic diversity due to its high replication rate, the error-prone RT and the recombination events between HIV-1 variants occurring during the viral replication after co-infection and/or superinfection events [22]–[24]. A large number of HIV-1 variants have been described based on viral sequences homology and HIV-1 has been divided into four groups: M (main), O (outlier), N (non-M, non-O) and P [23]. HIV-1 Group M is subdivided into 9 subtypes (A–D, F–H, J, K), at least 58 circulating recombinant forms (CRF) (http://www.hiv.lanl.gov/content/sequence/HIV/CRFs/CRFs.html) -designated by a number and the genetic subtypes present in their genome- and multiple unique recombinant forms (URF), widely spread throughout the world and with different recombination breakpoints from those found in CRFs. At least 20% of the 34 million infected humans have an inter-subtype URF or CRF [25] and new inter-subtype recombinants have increasing prevalence and complexity in the pandemic, including in some European countries [26]. Genetic variability in PR and CS provide the potential to modulate PR activity and susceptibility to PI [20]. For instance, CS polymorphisms in certain HIV-1 group M variants can influence the virological outcome of a first-line LPV/r single drug regimen [19].

Despite the high biological relevance of CS during HIV-1 maturation and the importance of the knowledge of CS conservation for the design of both novel antiretroviral agents acting as maturation inhibitors as well as for vaccine targeting CS in future, scarce data of HIV-1 variant-associated CS variability is available. Previous reports only analyzed a limited number of HIV-1 variants and site sequences [3], [27], [28]. Thus, the goal of our descriptive analysis was to analyze, for the first time, the conservation rate at amino acid level of each individual protease CS located within Gag or Pol for all HIV-1 groups, Group M subtypes and recombinants circulating in the HIV/AIDS pandemic. For this purpose we used a large dataset of HIV-1 sequences routinely deposited at Los Alamos National Center for Biotechnology Information or GenBank. We also defined the consensus sequences at each CS in all HIV-1 variant, identifying the highly conserved amino acids residues in each CS.

## Methods

### Sequence Data

**Figure 1:**
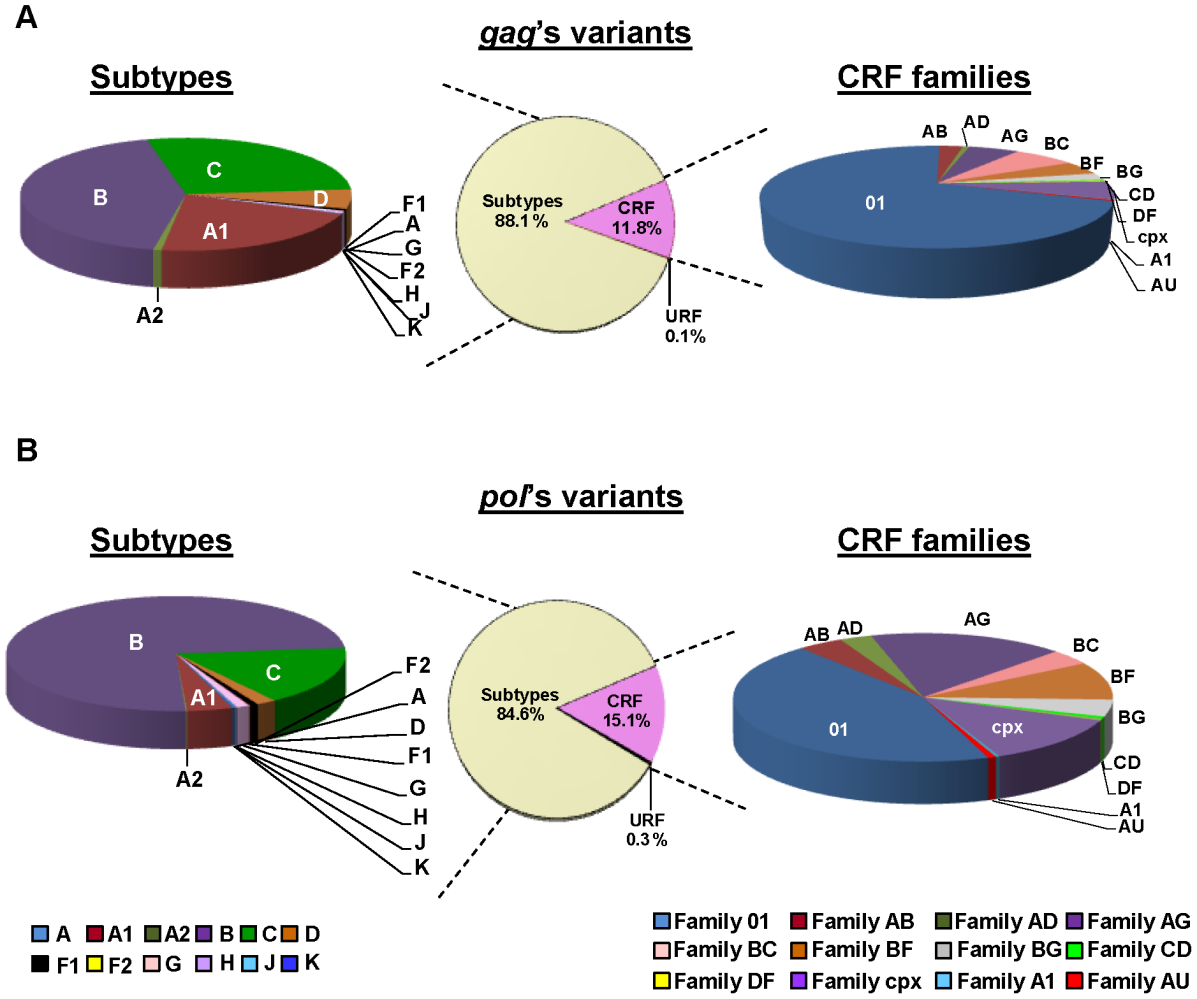
Distribution of HIV-1 Group M subtypes and CRF families. A total of 12,848 HIV-1 Group M sequences were retrieved from GenBank: 8,985 *gag* (A) and 3,863 *pol* (B) sequences.

**Figure 2:**
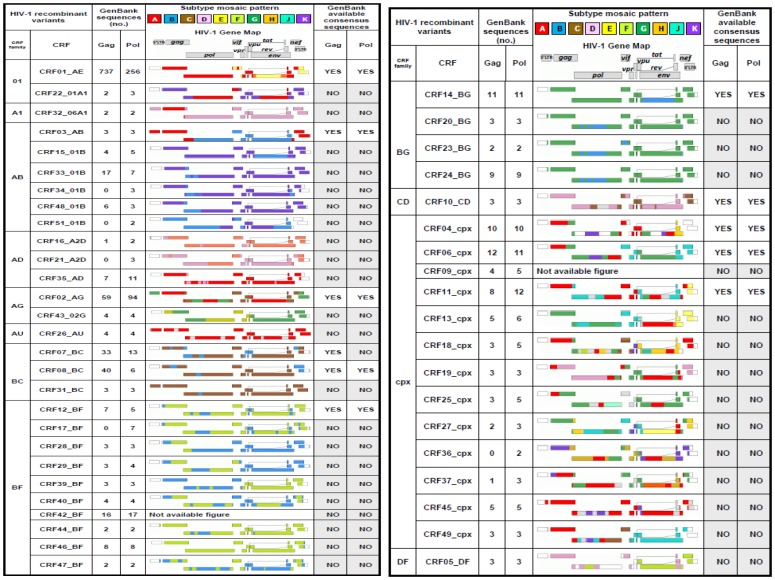
Gag and Pol HIV-1 recombinants sequences grouped by families. Availability of consensus sequences at GenBank. CRF sequences were grouped in 12 recombinant families; no, number; CRF, circulating recombinant forms http://www.hiv.lanl.gov/content/sequence/HIV/CRFs/CRFs.html; URF, unique recombinant forms. Other variants with consensus sequences from GenBank were: A1, A2, B, C, D, F1, G, H and K subtypes for *gag* and: A1, A2, B, C, D, F1, F2, G and H subtypes for *pol*. http://www.hiv.lanl.gov/content/sequence/NEWALIGN/align.html.

All the available HIV-1 *gag/pol* sequences were retrieved from GenBank, (http://www.ncbi.nlm.nih.gov/). The 12,934 *gag/pol* sequences comprised 2,844 nucleotides, located from 790 to 2,292 in *gag* and from 2,253 to 5,096 in *pol* encoding the proteins shown in **Table 1**. These sequences belonged to 4 groups (M, O, N, P), 9 Group M subtypes (A: sub-subtypes A1 and A2, B, C, D, F: sub-subtypes F1 and F2, G, H, J and K), 51 of the 58 CRF currently described, and with available sequences at GenBank and URF (**Figure 1**). For the subsequent analysis, we grouped in 12 recombinant families the closely related CRF sharing the same parental subtypes and very similar recombination patterns (**Figure 2**), as previously recommended [23]. All *gag/pol* nucleotides sequences were retrieved in FASTA format, including the subtype B HXB2 reference sequence. The MEGA (Molecular Evolutionary Genetics Analysis. Arizona States University, Tempe) program version 5.05 (http://www.megasoftware.net/) [29] was used to perform the nucleotides alignments and to translate them into amino acids.

**Table 1:** Gag and Pol HIV-1 proteins numbered in HXB2 genome.

		Gene		Protein	
HIV-1 proteins		Length (nucleotide)	Position (nucleotide)	Length (amino acid)	Position (amino acid)
**Gag**	**P17**	396	790–1185	132	263–394
	**P24**	693	1186–1878	231	395–625
	**P2**	42	1879–1920	14	626–639
	**P7**	165	1921–2085	55	640–694
	**P1**	48	2086–2133	16	695–710
	**P6**	156	2134–2289	52	711–762
	**Total**	**1500**	**790–2289**	**500**	**263–762**
**Pol**	**PR**	297	2253–2549	99	751–849
	**RT^p51^**	1320	2550–3869	440	850–1289
	**RT^p66^**	360	3870–4229	120	1290–1409
	**IN**	864	4230–5093	288	1410–1697
	**Total**	**2841**	**2253–5093**	**947**	**751–1697**

Nucleotides and amino acids numbered according to HXB2 subtype B reference strain (GenBank accession number K03455). P17, matrix; P24, capsid; P2, spacer peptide 1; P7, nucleocapside; P1, space peptide 2; PR, protease; RT, retrotranscriptase; IN, integrase; TFP, transframe protein. Retrieved from http://www.hiv.lanl.gov/.

### Identification of *gag* and *pol* Coding Regions and CS Sequences Defined at GenBank

After performing the alignments, we determined the residues and their location in Gag and Pol proteins (**Table 1**), identifying their nucleotides and amino acids and numbering them according to HXB2 subtype B reference strain (GenBank accession number K03455). We then identified the residues and the location of 11 cleavage sites (CS) within Gag and GagPol precursors: P17/P24, P24/P2, P2/P7, P7/P1, P1/P6*^gag^*, P7/TFP, TFP/P6*^pol^*, P6*^pol^*/PR, PR/RT^p51^, RT^p51^/RT^p66^ and RT^p66^/IN according to HXB2 sequence.

### Inferred Consensus Sequences

**Figure 3:**
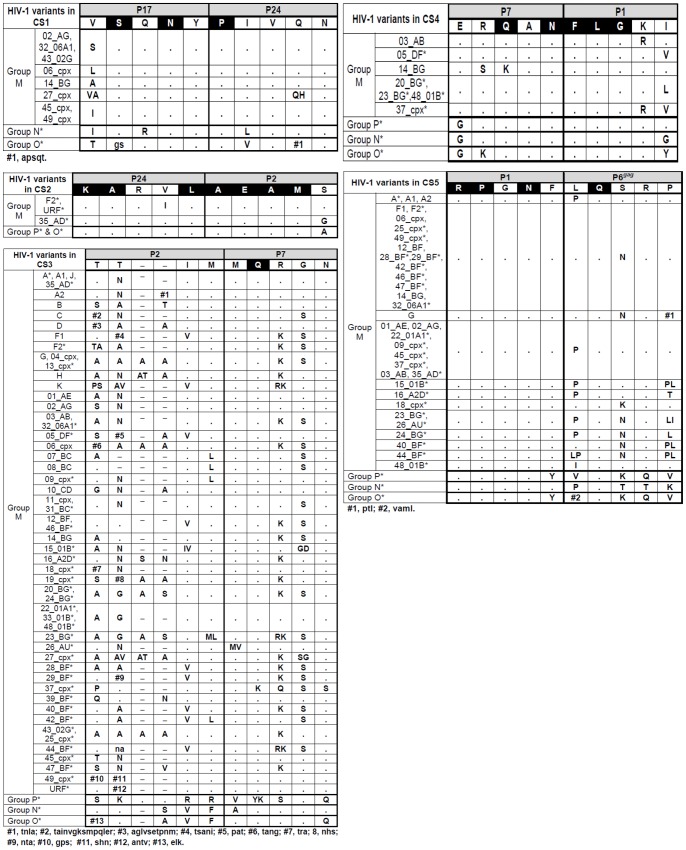
HIV-1 variants showing differences in CS1–CS5 amino acid *vs*. consensus-of-consensuses sequence from GenBank. Changes are only indicated when they appeared in a specific position in at least 50% of the GenBank downloaded sequences in order to compare them with the GenBank consensus-of-consensuses sequence. Asterisks indicate the HIV-1 variants shown in **Figure 2** with non available consensus sequence in GenBank. Black represents highly conserved amino acid residues and present in more than 99% of the 9,028 Gag and 3,906 GagPol HIV-1 sequences with respect to the consensus-of-consensuses sequence. When two residues within the analyzed sequences from each HIV-1 variant showed a conservation of 50% the two code letters were written in the upper case. When 3 or more residues appear in the same position and none presented a conservation of more than 50%, they were shown in lower case letters, which represented higher to lower conservation.

The consensus sequence is considered the sequence carrying the most frequent residues, either nucleotides or amino acids, at each position in a multiple sequence alignment. We collected all Gag and Pol consensus sequences available in GenBank (http://www.hiv.lanl.gov/content/sequence/NEWALIGN/align.html). The HIV-1 Group M variants with inferred consensus sequences in GenBank are indicated in **Figure 2**, and were calculated as explained in http://www.hiv.lanl.gov/content/sequence/NEWALIGN/align.html#consensus. Using our amino acid alignment, composed of 12,934 sequences, we determined new consensus sequences for each HIV-1 group and each Group M subtype, CRF and URF in the 11 CS (**Figures 3** **and** **4**). Then, we manually compared our inferred variant-associated consensus sequences at each CS with the ones provided by GenBank when available, showing the discrepancies.

**Figure 4:**
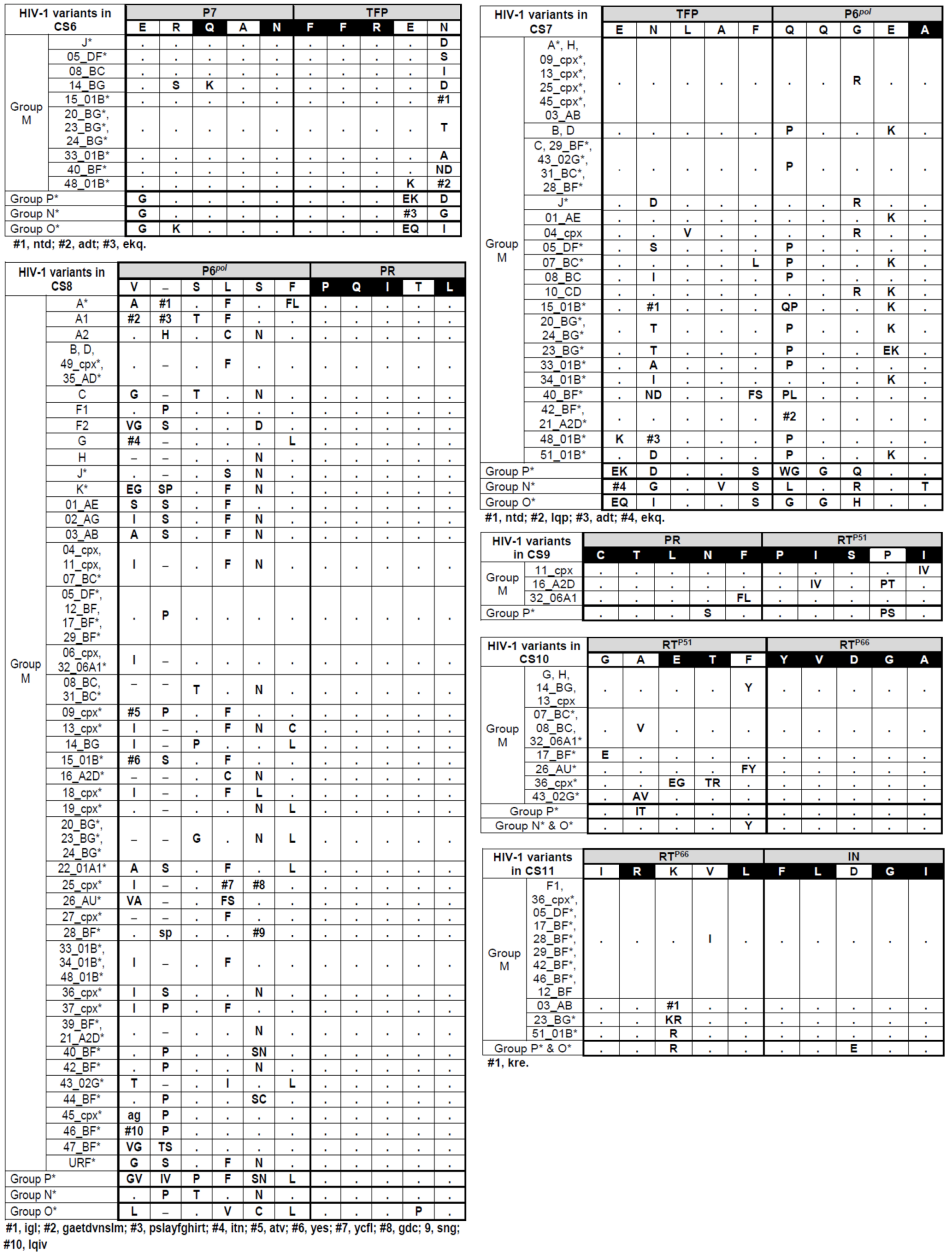
HIV-1 variants showing differences in CS6-CS11 amino acid *vs*. consensus-of-consensuses sequence from GenBank. See legend of **Figure 3**.

**Figure 5:**
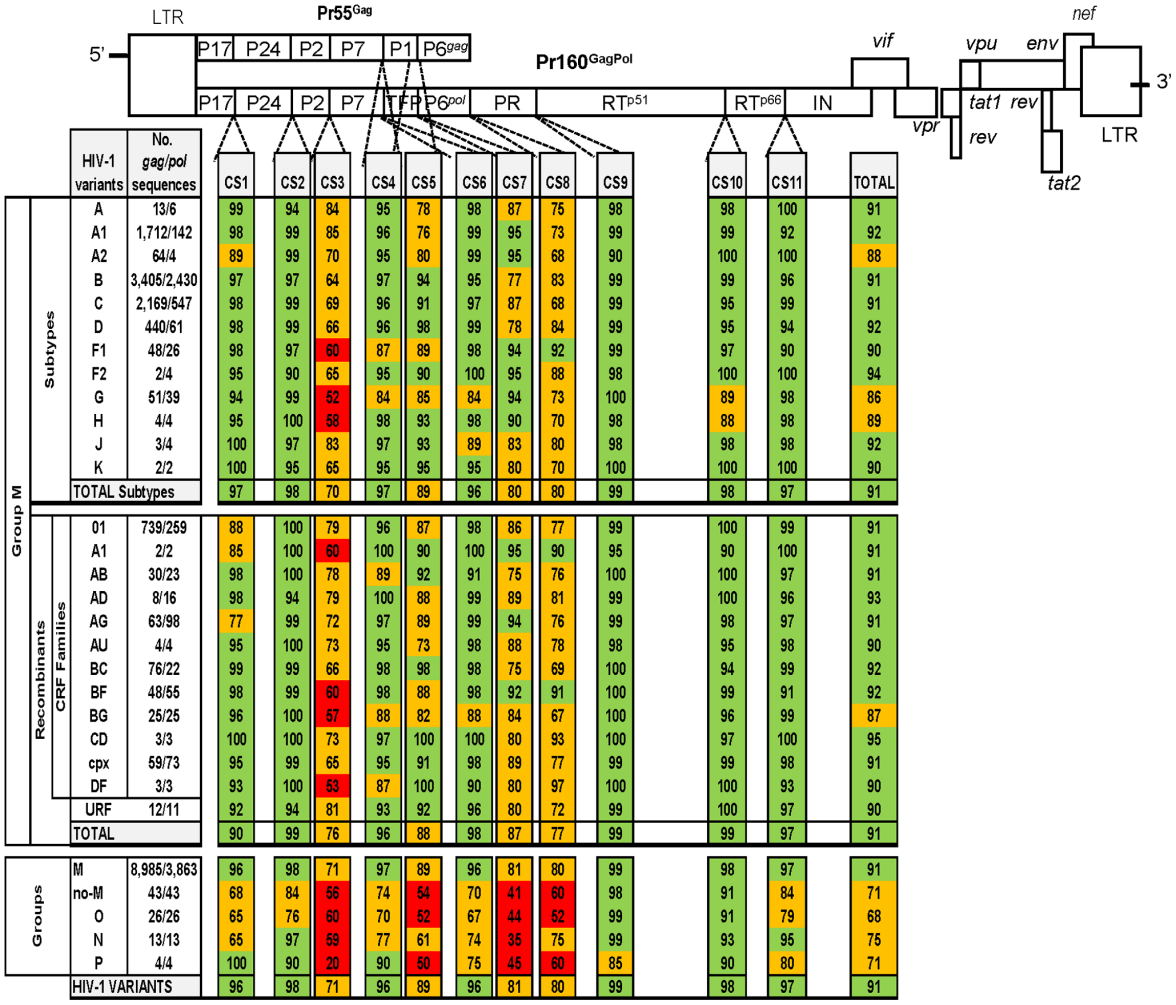
Amino acids CS conservation located in Gag and GagPol precursors in all HIV-1 variants. The conservation was determined by comparing our inferred consensus sequences with sequences from each HIV-1 variant *vs*. consensus-of-consensuses sequence retrieved from GenBank. Coloured boxes indicate the CS conservation rate at amino acid level: green (≥90%), orange (51–89%) and red (≤50%). The number in each coloured box shows the rate of conserved amino acid in each CS in all sequences of the corresponding HIV-1 variant. CS, cleavage site; P17, matrix; P24, capsid; P2, spacer peptide 1; P7, nucleocapside; P1, space peptide 2; TFP, transframe protein; PR, protease; RT, retrotranscriptase; IN, integrase; CRF, circulant recombinant form; URF, unique recombinant form.

We also retrieved the consensus-of-consensuses sequence provided by GenBank in order to generate an alignment of *gag* and *pol* individual consensus sequences that were used to analyze the conservation rate across sites and HIV-1 variants (**Figure 5**).

### Amino Acid Conservation Rate at CS Across HIV-1 Variants

All *gag* and *pol* sequences from GenBank were grouped according to the HIV-1 variant. We manually compared the degree of amino acid conservation in each CS, determined by the number of coincident amino acids among the 10 residues of each CS, in all downloaded sequences from each given variant with respect to the consensus-of-consensuses sequence provided by GenBank. The exact percentage of conserved amino acid residues for each HIV-1 variant and site with respect to the GenBank consensus-of-consensuses amino acid sequence was calculated counting the number of coincident residues in each of the 10 positions in the site in all sequences ascribed to a given variant divided by the total number of retrieved sequences for each variant and multiplied by the 10 residues of the site, expressing the result in percentages. To clarify results, we established a color code to show the conservation level in each site and HIV-1 variant (**Figure 5**).

### Data Analysis

Changes in rates were assessed using the chi-square analysis. Statistical analyses were performed using Epi Info v6.0 (Centers for Disease Control and Prevention, Atlanta, GA, USA). Significance was set at p<0.05.

## Results

### Gag/Pol HIV-1 Sequences Used for the Analysis and Variants Distribution

A total of 9,028 Gag and 3,906 Pol HIV-1 sequences were downloaded from GenBank database. They included 43/43 Gag/Pol sequences from 3 HIV-1 Groups (O, N, P) and 8,985/3,863 Gag/Pol sequences from Group M. **Figure 1** shows the Group M variants distribution of our retrieved sequence dataset, including a total of 7,913/3,269 Gag/Pol sequences from 9 HIV-1 group M subtypes (A: subsubtypes A1 and A2, B, C, D, F: sub-subtypes F1 and F2, G, H J and K), 1,060/583 Gag/Pol sequences ascribed to 51 CRF and 12/11 Gag/Pol URF sequences.

In order to simplify the analysis, we grouped all the sequences from the 51 CRFs in 12 different CRF families according to a similar recombination pattern (**Figure 2**). The downloaded sequences for each subtype and CRF family are detailed in **Figure 5**. Despite the large difference in the number of 8,985 Gag/3,863 Pol retrieved sequences, the specific distribution of HIV-1 Group M subtypes and CRF families was similar for both genes (**Figure 1**). Recombinants displayed 11.9% *gag* and 15.4% *pol* sequences. Among subtypes, sequences from subtype B were the most represented in both *gag/pol* (43%/74.3%) coding regions, followed by sequences ascribed to subtype C (27.4%/16.7%), sub-subtype A1 (21.6%/4.3%) and subtype D (5.6%/1.9%). There were no *gag* sequences from sub-subtype F2 and subtypes J and K available in our dataset. Within the recombinants, family 01 (69.7%/44.4%) was the most represented, followed by families BC (7.2%), AG (5.9%), cpx (5.6%) and BF (4.5%) in *gag* and by families AG (16.8%), cpx (12.5%), BF (9,4%), and BG (4.3%) in *pol*, among others. URF sequences represented less than 0.3% of downloaded sequences (12 *gag* and 11 *pol* sequences).

### HIV-1 Variant-specific *gag/pol* Consensus Sequences Available at GenBank

**Figure 2** shows the specific subtypes and recombinants with consensus sequences in *gag* and *pol* available in GenBank, which carries the most frequent residue, either nucleotide or amino acid, at each position in a multiple sequence alignment. **Table 2** summarizes the amino acids involved in each of the 11 CS (10 amino acids per site) in the HXB2 isolate as well as the consensus-of-consensuses sequence for each CS, defined by GenBank after the alignment of 28 *gag*/24 *pol* individual consensus sequences, corresponding to 8/7 subtypes among 9 in Group M and to 11/10 CRF within the 58 described (**Figure 2**). The consensus-of-consensuses sequence was taken as reference for the analysis of the conservation at amino acid level across variants in the 110 residues (10 amino acids in each of the 11 CS), as described in Methods.

**Table 2:** Gag and Pol HIV-1 CS numbered in HXB2 genome.

HIV-1				Gene		Protein	
No.	Name	Consensus-of-consensusesequence from GenBank	HXB2 sequence	Length (nucleotide)	Position (nucleotide)	Length (amino acid)	Position (amino acid)
**#1**	**P17/P24**	VSQNY/PIVQN	VSQNY/PIVQN	30	1171–1200	10	390–399
**#2**	**P24/P2**	KARVL/AEAMS	KARVL/AEAMS	30	1864–1893	10	621–630
**#3**	**P2/P7**	TT-IM/MQRGN	SATIM/MQRGN	30	1906–1935	10	635–644
**#4**	**P7/P1**	ERQAN/FLGKI	ERQAN/FLGKI	30	2071–2100	10	690–699
**#5**	**P1/P6** ***^gag^***	RPGNF/LQSRP	RPGNF/LQSRP	30	2119–2148	10	706–715
**#6**	**P7/TFP**	ERQAN/FFREN	ERQAN/FFRED	30	2071–2100	10	690–699
**#7**	**TFP/P6** ***^pol^***	ENLAF/QQGEA	EDLAF/LQGKA	30	2094–2123	10	698–707
**#8**	**P6** ***^pol^*** **/PR**	VSLSF/PQITL	VSFNF/PQVTL	30	2238–2267	10	746–755
**#9**	**PR/RT^p51^**	CTLNF/PISPI	CTLNF/PISPI	30	2535–2564	10	845–854
**#10**	**RT^p51^/RT^p66^**	GAETF/YVDGA	GAETF/YVDGA	30	3855–3884	10	1285–1294
**#11**	**RT^p66^/IN**	IRKVL/FLDGI	IRKVL/FLDGI	30	4215–4244	10	1405–1414

Nucleotides and amino acids numbered according to HXB2 subtype B reference strain (GenBank accession number K03455). Pr160^GagPol^ includes CS #1 to 11 and Pr55^Gag^ includes CS #1 to 5. Underlined amino acids show the changes in the HXB2 sequence *vs.* the consensus-of-consensuses sequence from GenBank. P17, matrix; P24, capsid; P2, spacer peptide 1; P7, nucleocapside; P1, space peptide 2; PR, protease; RT, retrotranscriptase; IN, integrase; TFP, transframe protein; No., CS position in Gag and GagPol precursors; CS, cleavage site. Retrieved from http://www.hiv.lanl.gov/.

### New Inferred Consensus Sequence in HIV-1 Groups, Subtypes and Recombinant *vs*. that Provided by GenBank

Since *gag* and *pol* consensus sequences were not defined by GenBank in all HIV-1 subtypes and CRF, we deduced our personal consensus sequence for all HIV-1 variants using our generated alignment of 9,028 Gag and 3,906 Pol HIV-1 sequences. We determined that the rate of amino acid residues among the retrieved sequences coincided with the consensus-of-consensuses in the corresponding site. For the first time, we inferred the consensus sequence in each site for the different HIV-1 groups and for all subtypes, sub-subtypes and recombinants within Group M. **Figures 3** **and** **4** show the HIV-1 variants that carry amino acid differences with the corresponding consensus-of-consensuses sequence from GenBank in CS. We identified when our inferred consensus sequence presented the same amino acid residue as consensus-of-consensuses provided by GenBank. All discrepancies found between our inferred variant-specific CS consensus sequences with the consensus-of-consensuses provided by GenBank were also identified (see **Table S1**).

### Identification of Highly Conserved Residues at CS

Interestingly, we identified 52/110 (47.3%) amino acids conserved in more than 99% of the 9,028 Gag and 3,906 Pol HIV-1 sequences with respect to the consensus-of-consensuses sequence and these are marked in black in **Figures 3** **and** **4**. Thus, nearly half of the residues involved in the 11 CS could accept a different degree of variant-dependent variability. Among sites, PR/RT^p51^ presented the highest number of highly conserved residues (9/10), followed by RT^p51^/RT^p66^ and P24/P2 (7/10), RT^p66^/IN (6/10), P7/P1 and P7/TFP (5/10), P1/P6*^gag^* and P6*^pol^*/PR (4/10), P17/P24 (3/10) and TFP/P6*^pol^* and P2/P7 (1/10).

### Observed Differences in CS Conservation Rates Across HIV-1 Variants and Sites

We evaluated the percentage of conserved residues in the retrieved sequences for each HIV-1 variant and site with respect to the GenBank consensus-of-consensuses amino acid sequence, as explained in Methods. We established a color code to indicate the different levels of conservation, and the exact amino acid conservation rate in each CS and variant (**Figure 5**). Interestingly, despite the structural and functional roles of proteins in the viral cycle, we observed different conservation rates across the sites and HIV-1 variants.

### Conservation Among CS

**Figure 6:**
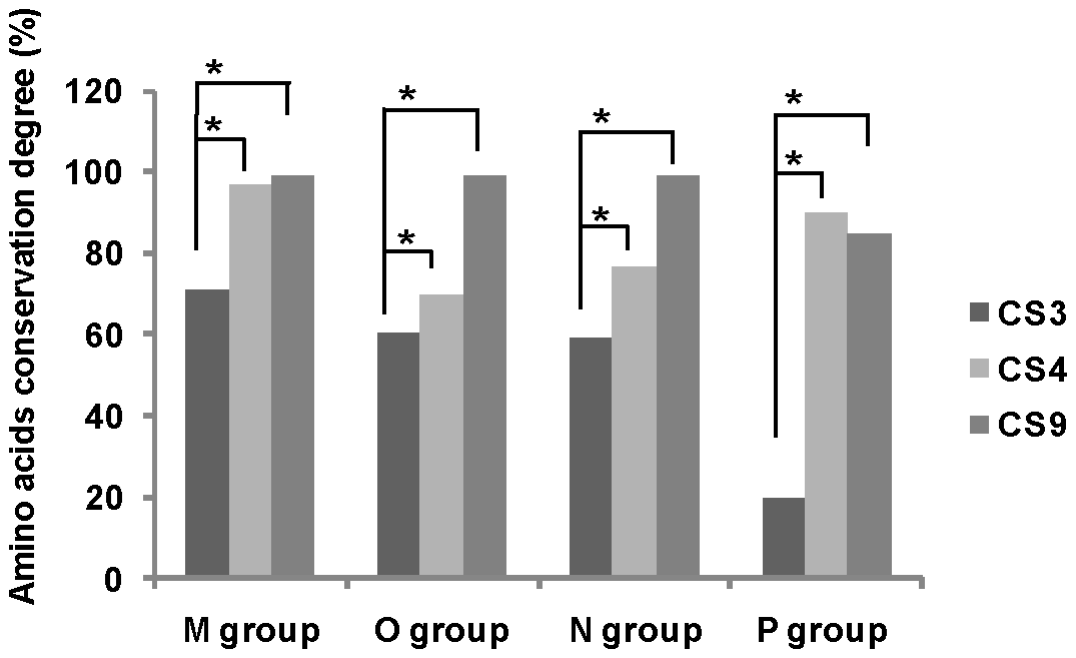
Conservation of the first and late processing sites at Gag and GagPol precursors. Late processing sites at Gag (CS4, P7/P1) and GagPol (CS9, PR/RT^p51^) precursors and first CS site (CS3, P2/P7) according to the CS order previously described [3], [5], [30]. *Significant difference, p<0.01.

In all HIV-1 variants, including all sequences ascribed to the 4 groups, we defined the conservation rate in each site (**Figure 5**). The CS with the highest number of conserved residues were CS9 (PR/RT^p51^, 99%), CS10 (RT^p51^/RT^p66^, 98%), CS2 (P24/P2, 98%), CS11 (RT^p66^/IN, 97%), CS1 (P17/P24, 96%), CS4 (P7/P1, 96%) and CS6 (P7/TFP, 96%). The least conserved CS across HIV-1 groups, Group M subtypes and recombinants were CS3 (P2/P7, 71%), CS8 (P6*^pol^*/PR, 80%), CS7 (TFP/P6*^pol^*, 81%) and CS5 (P1/P6*^gag^*, 89%). CS8 and CS3 showed different lengths across variants (data not shown). We observed a significantly higher conservation at the last processing sites in Gag (CS4, P7/P1) and GagPol (CS9, PR/RT^p51^) precursors compared to the first processing site (CS3, P2/P7) in the 4 HIV-1 groups (**Figure 6**), according to the proccessing order previously defined [3], [5], [30].

### Conservation among HIV-1 Groups

We observed differences in the CS conservation rate across HIV-1 groups and sites (**Figure 5**). Interestingly, CS10 (RT^p51^/RT^p66^) showed more than 90% of conserved residues regarding consensus-of-consensuses amino acid sequence in the 4 HIV-1 groups. Comparing M and non-M Groups, we observed higher conservation in CS9 (99% and 98%, respectively) and in CS10 (98% and 91%, respectively). However, CS7 (TFP/P6*^pol^*) presented the poorest conservation rate across non-M Groups (41%), followed by CS5 (P1/P6*^gag^*, 54%), CS3 (P2/P7, 56%), CS8 (P6*^pol^*/PR, 60%) and CS1 (P17/P24, 68%). Group O showed the lowest conservation in 6 of the 11 CS (CS1, CS2, CS4, CS6, CS8 and CS11), Group N in CS1 and CS7 and Group P in CS3, CS5, CS9 and CS10 (**Figures 3** **and** **4**). Considering the 11 CS, we found a significantly higher conservation rate among Group M *vs*. non-M Groups HIV-1 variants (91% *vs.* 71%, *p*<0.001), probably due to the use of group M consensus for comparison.

### Conservation among Group M Subtypes and Recombinants

Seven sites (CS1, CS2, CS4, CS6, CS9, CS10 and CS11) were well conserved within the total HIV-1 Group M subtypes and recombinants, showing more than 90% conservation (**Figure 5**). Four sites (CS3, CS5, CS7 and CS8) were more variable in a large number of HIV-1 variants. The lowest conservation rate in the 11 CS was found in the following HIV-1 Group M subtypes and recombinants: CS1 (P17/P24) in sub-subtype A2 (89%) and in AG recombinant family (77%); CS2 (P24/P2) in sub-subtype F2 (90%) and recombinant families AD and URF (both 94%); CS3 (P2/P7) in subtypes G (52%) and recombinant family DF (53%); CS4 (P7/P1) in subtype G (84%) and recombinant family DF (87%); CS5 (P1/P6*^gag^*) in sub-subtype A1 (76%) and recombinant family AU (73%); CS6 (P7/TFP) in subtype G (84%) and recombinant family BG (88%); CS7 (TFP/P6*^pol^*) in subtype B (77%) and recombinant families AB and BC (both 75%); CS8 (P6*^pol^*/PR) in sub-subtype A2 and subtype C (both 68%) and recombinant family BG (67%); CS9 (PR/RT^p51^) sub-subtype A2 (90%) and recombinant family A1 (95%); CS10 (RT^p51^/RT^p66^) in clades H (88%) and G (89%) and recombinant family A1 (90%); and CS11 (RT^p66^/IN) in sub-subtype F1 (90%) and recombinant family BF (91%). Thus, subtype G showed the highest variability in CS3, CS4 and CS6 and subtype B in CS7 compared to other Group M subtypes.

The recombinant families DF, BG, A1 and BF showed the highest variability in CS3 (**Figure 5**). The CS2, CS6, CS9, CS10 and CS11 were highly conserved (94%–99%) across all CRF families and URF. Overall, recombinants showed the same conservation as subtypes (91%) in the 11 analyzed CS, the BG recombinant family with 87%, subtype G with 86%, sub-subtype A2 with 88% and subtype H with 89%. The HIV-1 variants that presented the lowest CS conservation were significantly lower in subtypes *vs.* recombinants in CS3 (70% *vs.*76%, *p*<0.001) and in CS7 (80% *vs*. 87%, *p*<0.001) and higher in CS1 (97% *vs.* 90%, *p*<0.001).

### Conservation among Sub-subtypes in Specific Sites

Sub-subtype A2 presented significantly lower conservation than sub-subtype A1 in CS1 (89% *vs*. 98%, *p*<0.001), in CS3 (70% *vs.* 85%, *p*<0.001) and in CS9 (90% *vs.* 99%, *p*<0.001), significantly higher conservation in CS5 (80% *vs.* 76%, *p* = 0.04) and the conservation was of great significance in CS11 (100% *vs.* 92%, *p* = 0.06). Sub-subtype F2 showed superior conservation compared to sub-subtype F1 in CS11 (100% *vs.*90%, *p* = 0.04), although the number of available F2 sequences was very low (see **Figure 5**).

## Discussion

HIV-1 genomes analysis provides useful biological information in terms of the structure and function of viral proteins [31]. The correct core formation is essential for the production of infectious HIV particles and this is known to be dependent on accurate proteolytic processing of Gag. Thus, mutations that disrupt the cleavage of individual sites or alter the order in which sites are cleaved result in aberrant particles that have significantly reduced infectivity [6]. Although other publications previously reported that certain CS were more conserved than others, they only analyzed a very limited number of HIV-1 variants and site sequences [3], [27], [28]. Thus, to our knowledge, our study is the first to evaluate the conservation rate in 11 CS within Gag and GagPol precursors and to define the consensus sequences in each site using a large sequence dataset including all Group M subtypes and most CRF. Furthermore, it is the first study that includes sequences from Groups N, O and P, identifying completely conserved residues at CS present in all 4 groups. We showed the conservation rate in each HIV-1 variant and CS, finding different conservation rates across sites in the 4 HIV-1 groups and in Group M variants, including a complete panel of recombinants, whose prevalence and complexity is increasing in the pandemic [23]. In fact, the different clade distribution for *gag* and *pol* sequences retrieved for GenBank used in the study could be explained by the large number of recombinants circulating in pandemic, with different clades in different viral genome genes.

### New Findings on CS Variability Across HIV-1 Variants

Only a limited number of studies have previously evaluated the natural variation within *gag* and CS [3], [5], [12], [28], [32], [33]. However, these have mainly focused on subtypes B and/or C and they have analyzed a smaller dataset or a limited number of CS in most cases. Furthermore, the majority of the studies used HXB2 subtype B as reference sequence for conservation analysis [5], [32], [33], pNL-4-3subtype B [12] or the Group M most recent common ancestor sequence [3]. Only one used the consensus-of-consensuses sequence provided by GenBank as a reference for comparisons [28]. None inferred a consensus sequences for each analyzed HIV-1 variant and site. Other studies included either recombinants or non-M Group sequences. Despite the wide variety in the number of sequences that we downloaded from GenBank for Group M (8,985/3,863 *gag/pol* sequences) with respect to the rest of groups (43/43 *gag/pol* sequences) or certain subtypes (H, J, K), sub-subtypes (F2) or CRF, available data permitted the establishment of a comparison among conservation rates at CS and we were able to define specific-variant differences at each CS consensus sequence for each HIV-1 group, subtype, CRF and URF (see **Figure 5**). Our data reflects that the degree of conservation differs between individual amino acid positions at CS and provides significant discrepancies across specific HIV-1 variants and CS, thus improving the available GenBank data for specific-HIV-1 variants consensus sequences.

By using a large dataset of 12,934 sequences from all HIV-1 variants, our study revealed that the CS3, CS5, CS7 and CS8 were the least conserved processing sites across all HIV-1 variants. This finding is in agreement with previous publication using a smaller dataset with 32 subtype C, 34 subtype B and 18 other subtypes sequences [3]. Additional studies are necessary to understand the higher variability in these CS with important roles in the viral cycle. In more detail, CS3 is the first processing site in Gag and GagPol precursors and it is critical for RNA dimer maturation [34]; CS7 is involved in the activation of the viral PR and in the timing and specificity of GagPol cleavage [35]; CS5 is responsible for protein P6*^gag^* synthesis which is required for the mature and infectious virion release [36]; CS8 is essential for PR autoprocessing and, it could probably be involved in the correct required PR dimerization [37].

### Structural Constrains to CS Variation

Complex interactions of the substrate amino acids within the active site of the viral PR are required for efficient Gag and GagPol cleavage by the PR. HIV-1 PR is only functional in dimeric form and a single monomer is embedded within each precursor. Two individual monomers in different GagPol chains must, therefore, come together to form an embedded dimeric PR, which ultimately cleaves itself into a mature form [37]. HIV-1 maturation requires the recognition by PR of the asymmetric, three-dimensional conformation of the Gag substrate, rather than a particular peptide sequence [38] and, afterwards, PR mediates the cleavage of the HIV-1 structural Gag and GagPol polyproteins by interacting with specific CS [6], [39]. Each substrate has a unique structure that differs in amino acid composition [3]. It is thought that these small differences in substrate structure impact affinity for the viral PR and contribute to the highly regulated and ordered stepwise process of maturation in which the individual cleavages occur at different times and rates [3], [30]. Additional determinants beyond amino acid sequences and local secondary structures of CS are involved in Gag and GagPol processing [7]. As Gag is conserved, there are constraints on the viability of viral strains with multiple mutations due to the fact that combined mutations are likely to destabilize multiprotein structural interactions that are critical for viral function [40]. Thus, amino acid sequence conservation indicates that the specific amino acids are required to maintain basic structure and function, although other authors have suggested an important role of RNA structure in HIV-1 conservation [33], [41]. It is known that physicochemical and structural properties of certain HIV-1 proteins with functional roles in the viral cycle as gp41 can be strongly conserved despite substantial sequence diversity, apparently indicating a delicate balance between evolutionary pressures and the conservation of protein structure [42]. The protein structure, specifically α-helix domains, has been associated with conservation in HIV-1 [33] and is a stable structural element in proteins [43].

Our study reveals which can be the most important CS amino acid sequence for maintaining viral processing by PR and the level of tolerance to amino acid change in each HIV-1 variant. Moreover, the significantly higher conservation observed comparing the late *vs*. the first CS in Gag and GagPol precursors (flanking the PR) would suggest a higher requirement of structural constrains in the last steps of viral processing. Although the aim of our study is purely descriptive, we strongly believe that it can serve as a working tool for research into the better understanding of the CS structure required for a correct cleavage efficacy across HIV-1 variants and for the design of maturation inhibitors and vaccines targeting CS. Understanding HIV-1 *gag* and *pol* co-evolution [44], [45] and the influence of naturally occurring specific-variant polymorphisms at PR [46] in the cleavage process is also crucial for a better interpretation of the biological significance of amino acid changes in CS in the context of a specific HIV-1 variant. Lastly, whether or not the variant specific-residues located in each CS modulate the replicative capacity of the corresponding variant, as was observed for specific natural polymorphisms in the PR in some non-B variants [47], requires further investigation.

It has been suggested that sequences around the CS in Gag are equally conserved as functional motives and sequences targeted by RT inhibitors and are more conserved than non-functional motives [28]. These authors suggested that the amino acid sequences overlapping the CS are immunogenic and, consequently, a vaccine targeting CS could be used for the majority of the world population [28]. Thus, our data on CS conservation across HIV-1 variants could provide useful data to design potential targets for an effective vaccine development against HIV effective for all groups, subtypes and recombinants. Moreover, since mutations within CS have been associated with PI exposure and maturation inhibitor resistance [5], [32], our results could potentially provide a better understanding of the role of *gag* in antiretroviral resistance and in the development of future maturation inhibitors [4].

## Conclusion

This descriptive study firstly determines the CS conservation degree across most HIV-1 variants and sites in a large dataset composed of 12,934 sequences, inferring the consensus sequences at amino acid level in 11 CS in all Group M subtypes and most CRF and URF, as well as in Groups O, N and P. Our results provide new findings that can help for a better understanding of viral evolution, Gag and GagPol precursors’ processing and *gag* structure-function relationships, among others. Our descriptive research could help other researchers in the design of both novel antiretroviral agents acting as maturation inhibitors and for vaccine targeting CS. The biological significance of HIV-1 variant-associated variability found in each processing site in our study needs further future investigation.

## Supporting Information

Table S1 **HIV-1 variants showing differences with the CS consensus-of-consensuses sequence inferred by GenBank.**
(DOC)
