# Supplementary material for: New Findings in Cleavage Sites Variability across Groups, Subtypes and Recombinants of Human Immunodeficiency Virus Type 1
Source: PLoS One. 2014 Feb 7;9(2):e88099. doi: 10.1371/journal.pone.0088099 (PMC3917854; doi:10.1371/journal.pone.0088099)
Supplement: Table S1 — HIV-1 variants showing differences with the CS consensus-of-consensuses sequence inferred by GenBank. (DOC) [file pone.0088099.s001.doc]

**Tables S1. HIV-1 variants showing differences with the CS consensus-of-consensuses sequences inferred by GenBank.**

**Footnote Tables S1:** Amino acid substitutions with respect to the CS consensus-of-consensuses sequence are indicated when they appeared in a specific position in the GenBank consensus sequences in at least 50% of the 28/24 Gag/Pol sequences used by GenBank to infer each HIV-1 variant specific CS consensus sequence (<http://www.hiv.lanl.gov/content/sequence/NEWALIGN/align.html>). Discrepancies found between our inferred HIV-1 variant-specific CS consensus sequences shown in **Figure 3** of the manuscript compared with the consensus-of-consensuses sequences provided by GenBank for the same variant were also identified in grey. Dash represents an amino acid deletion in the corresponding position of variant consensus sequence.

| **HIV-1 variants in CS1** | | **P17** | | | | | **P24** | | | | |
| --- | --- | --- | --- | --- | --- | --- | --- | --- | --- | --- | --- |
|  |  | **V** | **S** | **Q** | **N** | **Y** | **P** | **I** | **V** | **Q** | **N** |
| Group M | A2 | **S** | **.** | **.** | **.** | **.** | **.** | **.** | **.** | **.** | **.** |
|  | 02_AG | **−** | **−** | **.** | **.** | **.** | **.** | **.** | **.** | **.** | **.** |
|  | 06_cpx | **L** | **.** | **.** | **.** | **.** | **.** | **.** | **.** | **.** | **.** |
|  | 14_BG | **A** | **.** | **.** | **.** | **.** | **.** | **.** | **.** | **.** | **.** |

| **HIV-1 variants in CS2** | | **P24** | | | | | **P2** | | | | |
| --- | --- | --- | --- | --- | --- | --- | --- | --- | --- | --- | --- |
|  |  | **K** | **A** | **R** | **V** | **L** | **A** | **E** | **A** | **M** | **S** |
| Group M | K | **.** | **.** | **.** | **I** | **.** | **.** | **.** | **.** | **.** | **.** |

| **HIV-1 variants in CS3** | | **P2** | | | | | | **P7** | | | | |
| --- | --- | --- | --- | --- | --- | --- | --- | --- | --- | --- | --- | --- |
|  |  | **T** | **T** | **−** | **−** | **I** | **M** | **M** | **Q** | **R** | **G** | **N** |
| Group M | A1, 02_AG | **.** | **N** | **.** | **.** | **.** | **.** | **.** | **.** | **.** | **.** | **.** |
|  | A2 | **.** | **N** | **T** | **N** | **.** | **.** | **.** | **.** | **.** | **.** | **.** |
|  | B | **S** | **A** | **.** | **T** | **.** | **.** | **.** | **.** | **.** | **.** | **.** |
|  | C, 11_cpx | **.** | **N** | **.** | **.** | **.** | **.** | **.** | **.** | **.** | **S** | **.** |
|  | D | **S** | **A** | **A** | **.** | **V** | **.** | **.** | **.** | **.** | **.** | **.** |
|  | F | **.** | **A** | **.** | **.** | **.** | **.** | **.** | **.** | **.** | **.** | **.** |
|  | F1 | **.** | **.** | **.** | **.** | **.** | **.** | **.** | **.** | **K** | **S** | **l** |
|  | G, 04_cpx | **A** | **A** | **A** | **A** | **.** | **.** | **.** | **.** | **K** | **S** | **.** |
|  | H | **A** | **N** | **A** | **A** | **.** | **.** | **.** | **.** | **K** | **.** | **.** |
|  | K | **.** | **A** | **.** | **.** | **V** | **.** | **.** | **.** | **.** | **.** | **.** |
|  | 01_AE | **A** | **N** | **.** | **.** | **.** | **.** | **.** | **.** | **.** | **.** | **.** |
|  | 03_AB | **A** | **N** | **.** | **.** | **.** | **.** | **.** | **.** | **K** | **S** | **.** |
|  | 06_cpx | **.** | **E** | **A** | **A** | **.** | **.** | **.** | **.** | **K** | **S** | **.** |
|  | 07_BC, 08_BC | **.** | **−** | **.** | **.** | **.** | **L** | **.** | **.** | **.** | **S** | **.** |
|  | 10_CD | **A** | **N** | **A** | **.** | **.** | **.** | **.** | **.** | **.** | **.** | **.** |
|  | 12_BF | **.** | **.** | **.** | **.** | **V** | **.** | **.** | **.** | **K** | **S** | **.** |
|  | 14_BG | **A** | **.** | **.** | **.** | **.** | **.** | **.** | **.** | **K** | **S** | **.** |

| **HIV-1 variants in CS4** | | **P7** | | | | | **P1** | | | | |
| --- | --- | --- | --- | --- | --- | --- | --- | --- | --- | --- | --- |
|  |  | **E** | **R** | **Q** | **A** | **N** | **F** | **L** | **G** | **K** | **I** |
| Group  M | 03_AB | **.** | **.** | **.** | **.** | **.** | **.** | **.** | **.** | **R** | **.** |
|  | 04_cpx | **.** | **.** | **.** | **.** | **.** | **.** | **.** | **.** | **R** | **M** |
|  | 14_BG | **.** | **S** | **K** | **.** | **.** | **.** | **.** | **.** | **.** | **.** |

| **HIV-1 variants in CS5** | | **P1** | | | | | **P6*^gag^*** | | | | |
| --- | --- | --- | --- | --- | --- | --- | --- | --- | --- | --- | --- |
|  |  | **R** | **P** | **G** | **N** | **F** | **L** | **Q** | **S** | **R** | **P** |
| Group M | A1, 01_AE, 02_AG, 03_AB. | **.** | **.** | **.** | **.** | **.** | **P** | **.** | **.** | **.** | **.** |
|  | A2 | **.** | **.** | **.** | **.** | **.** | **P** | **.** | **.** | **.** | **T** |
|  | C, G, 06_cpx, 12_BF, 14_BG | **.** | **.** | **.** | **.** | **.** | **.** | **.** | **N** | **.** | **.** |

| **HIV-1 variants in CS6** | | **P7** | | | | | **TFP** | | | | |
| --- | --- | --- | --- | --- | --- | --- | --- | --- | --- | --- | --- |
|  |  | **E** | **R** | **Q** | **A** | **N** | **F** | **F** | **R** | **E** | **N** |
| Group M | 14_BG | **.** | **S** | **K** | **.** | **.** | **.** | **.** | **.** | **.** | **.** |

| **HIV-1 variants in CS7** | | **TFP** | | | | | **P6*^pol^*** | | | | |
| --- | --- | --- | --- | --- | --- | --- | --- | --- | --- | --- | --- |
|  |  | **E** | **N** | **L** | **A** | **F** | **Q** | **Q** | **G** | **E** | **A** |
| Group M | A2, H, 03_AB | **.** | **.** | **.** | **.** | **.** | **.** | **.** | **R** | **.** | **.** |
|  | B | **.** | **D** | **.** | **.** | **.** | **P** | **.** | **.** | **K** | **.** |
|  | C | **.** | **.** | **.** | **.** | **.** | **P** | **.** | **.** | **.** | **.** |
|  | D | **.** | **.** | **.** | **.** | **.** | **P** | **.** | **.** | **K** | **.** |
|  | 01_AE | **.** | **.** | **.** | **.** | **.** | **.** | **.** | **.** | **K** | **.** |
|  | 04_cpx | **.** | **.** | **V** | **.** | **.** | **.** | **.** | **R** | **.** | **.** |
|  | 08_BC | **.** | **I** | **.** | **.** | **.** | **P** | **.** | **.** | **.** | **.** |
|  | 10_CD | **.** | **.** | **.** | **.** | **.** | **.** | **.** | **R** | **K** |  |

| **HIV-1 variants in CS8** | | **P6*^pol^*** | | | | | | **PR** | | | | |
| --- | --- | --- | --- | --- | --- | --- | --- | --- | --- | --- | --- | --- |
|  |  | **V** | **−** | **S** | **L** | **S** | **F** | **P** | **Q** | **I** | **T** | **L** |
| Group M | A1 | **G** | **P** | **T** | **F** | **.** | **.** | **.** | **.** | **.** | **.** | **.** |
|  | A2 | **.** | **H** | **.** | **C** | **N** | **.** | **.** | **.** | **.** | **.** | **.** |
|  | B | **.** | **−** | **.** | **F** | **.** | **.** | **.** | **.** | **.** | **.** | **.** |
|  | C | **L** | **−** | **T** | **.** | **N** | **.** | **.** | **.** | **.** | **.** | **.** |
|  | D | **.** | **−** | **.** | **F** | **N** | **.** | **.** | **.** | **.** | **.** | **.** |
|  | F1, 12_BF | **.** | **P** | **.** | **.** | **.** | **.** | **.** | **.** | **.** | **.** | **.** |
|  | F2 | **G** | **S** | **.** | **.** | **D** | **.** | **.** | **.** | **.** | **.** | **.** |
|  | H | **−** | **−** | **.** | **.** | **.** | **.** | **.** | **.** | **.** | **.** | **.** |
|  | G, 06_cpx | **I** | **−** | **.** | **.** | **.** | **.** | **.** | **.** | **.** | **.** | **.** |
|  | 01_AE | **S** | **S** | **.** | **F** | **.** | **.** | **.** | **.** | **.** | **.** | **.** |
|  | 02_AG | **I** | **S** | **.** | **F** | **N** | **.** | **.** | **.** | **.** | **.** | **.** |
|  | 03_AB | **A** | **S** | **.** | **F** | **N** | **.** | **.** | **.** | **.** | **.** | **.** |
|  | 04_cpx, 11_cpx | **I** | **−** | **.** | **F** | **N** | **.** | **.** | **.** | **.** | **.** | **.** |
|  | 08_BC | **−** | **−** | **T** | **.** | **N** | **.** | **.** | **.** | **.** | **.** | **.** |
|  | 14_BG | **I** | **−** | **P** | **.** | **.** | **L** | **.** | **.** | **.** | **.** | **.** |

| **HIV-1 variants in CS10** | | **RT^P51^** | | | | | **RT^P66^** | | | | |
| --- | --- | --- | --- | --- | --- | --- | --- | --- | --- | --- | --- |
|  |  | **G** | **A** | **E** | **T** | **F** | **Y** | **V** | **D** | **G** | **A** |
| Group M | G, H, 14_BG | **.** | **.** | **.** | **.** | **Y** | **.** | **.** | **.** | **.** | **.** |
|  | 08_BC | **.** | **V** | **.** | **.** | **.** | **.** | **.** | **.** | **.** | **.** |

| **HIV-1 variants in CS11** | | **RT^P66^** | | | | | **IN** | | | | |
| --- | --- | --- | --- | --- | --- | --- | --- | --- | --- | --- | --- |
|  |  | **I** | **R** | **K** | **V** | **L** | **F** | **L** | **D** | **G** | **I** |
| Group M | F1, 12_BF | **.** | **.** | **.** | **I** | **.** | **.** | **.** | **.** | **.** | **.** |
